# A linear programming-based strategy to save pipette tips in automated DNA assembly

**DOI:** 10.1093/synbio/ysac004

**Published:** 2022-04-11

**Authors:** Kirill Sechkar, Zoltan A Tuza, Guy-Bart Stan

**Affiliations:** Department of Bioengineering, Imperial College London, London SW7 2AZ, UK; Department of Bioengineering, Imperial College London, London SW7 2AZ, UK; Department of Bioengineering, Imperial College London, London SW7 2AZ, UK

**Keywords:** DNA assembly, pipette tip consumption minimization, automation, linear programming

## Abstract

Laboratory automation and mathematical optimization are key to improving the efficiency of synthetic biology research. While there are algorithms optimizing the construct designs and synthesis strategies for DNA assembly, the optimization of how DNA assembly reaction mixes are prepared remains largely unexplored. Here, we focus on reducing the pipette tip consumption of a liquid-handling robot as it delivers DNA parts across a multi-well plate where several constructs are being assembled in parallel. We propose a linear programming formulation of this problem based on the capacitated vehicle routing problem, as well as an algorithm which applies a linear programming solver to our formulation, hence providing a strategy to prepare a given set of DNA assembly mixes using fewer pipette tips. The algorithm performed well in randomly generated and real-life scenarios concerning several modular DNA assembly standards, proving to be capable of reducing the pipette tip consumption by up to $59\%$ in large-scale cases. Combining automatic process optimization and robotic liquid handling, our strategy promises to greatly improve the efficiency of DNA assembly, either used alone or combined with other algorithmic DNA assembly optimization methods.

Graphical Abstract

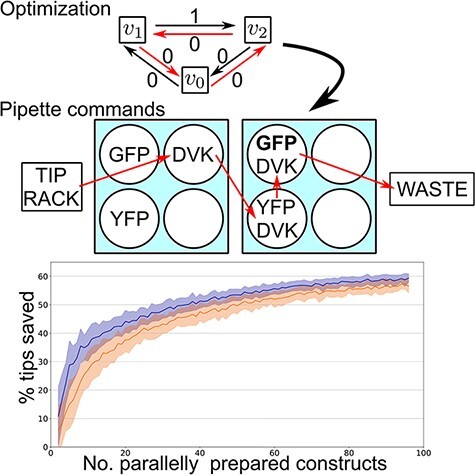

## 1. Introduction

Among the key factors driving synthetic biology research, which is concerned with efficiently engineering organisms with useful new properties, is the automation of laboratory processes. Allowing to produce much larger and higher-quality experimental datasets, it not only accelerates the rate of research, but also enables the use of computational analysis tools that require large amounts of experimental data, which are difficult to produce in a reasonable time by low-throughput human manual labor [1].

Implementing a genetic circuit in living organisms typically involves DNA assembly—the joining of DNA fragments (‘parts’), where each part is usually a functional genetic element, into a longer nucleic acid chain (‘construct’), which can then be introduced into a host organism to induce the desired phenotype. Likewise to other aspects of synthetic biology research, DNA assembly has been greatly impacted by the adoption of laboratory automation practices—namely, the use of liquid-handling robots [2]. A particular advantage of using such robots is the parallel preparation of multiple different constructs in distinct wells of a single multi-well array plate, which greatly improves the throughput and time efficiency of DNA assembly.

Metrics quantifying the cost and time benefits of executing a given DNA assembly scenario by a liquid-handling robot instead of a human worker have been proposed [3]. Furthermore, recent years have seen the rise of algorithms aiming to improve the efficiency of automated DNA assembly. Primarily, they deal with the design stage of the research cycle: for example, the number of reactions required to obtain the desired construct in a multistep assembly can be minimized by representing possible sequences of assembly steps as graphs and selecting the optimal one [4]. Alternatively, optimization algorithms may determine the set of DNA fragments from which the desired construct can be assembled most efficiently. These fragments can also be codon optimized to increase gene expression and facilitate DNA assembly by tuning the nucleotide sequence’s GC content. Thus, a trade-off between the assembly’s design requirements and the ease of DNA manufacturing can be found algorithmically [5]. Meanwhile, lower-level optimization of a liquid-handling robot’s exact actions as it sets up DNA assembly mixes (e.g. when several constructs are made in parallel) has largely remained neglected. This contrasts with the general practice of process automation, where compilers optimize the commands and execution order of human-written programs on several levels at once [6].

Many of the widely adopted DNA assembly standards (e.g. BASIC [2], MoClo [7] or Start–Stop assembly [8]) are ‘one-pot’, which means that a DNA construct is produced by mixing all of its parts together, so that they autonomously assemble into the desired construct. Therefore, setting up parallel DNA assembly reactions on a well plate involves preparing different mixtures of several DNA parts in different wells. Although in the case of parallel one-pot assemblies a single part is most often required in multiple distinct constructs, reusing the same pipette tip to deliver a part to several wells introduces a high risk of contaminating a master mix by the DNA parts present in the previously visited wells. Therefore, usually either a new tip is used for delivery to each well, which results in an excessively large uptake of pipette tips and thus increased operating costs, or the pipette is washed after every delivery, which can be time-consuming.

**Figure 1.**
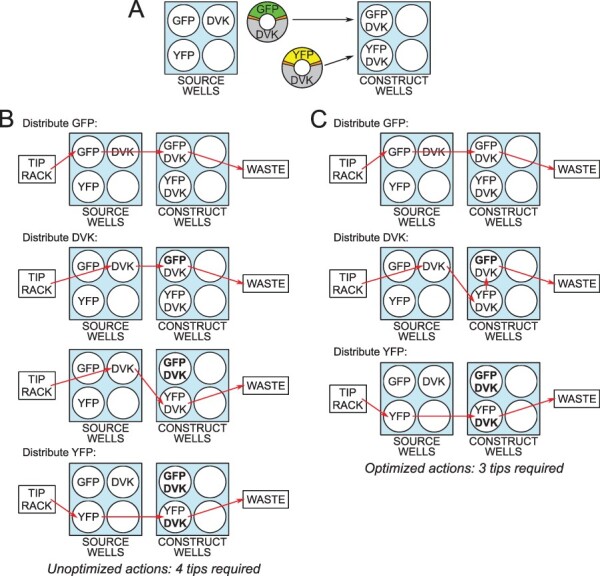
An instance of pipette tip consumption minimization. (A) One construct comprises the Green Fluorescent Protein (GFP) gene inserted into the DVK vector backbone, while the other has the same backbone hosting the Yellow Fluorescent Protein (YFP) gene instead. (B) Unoptimized sequence of DNA part additions. Source wells display which DNA part solution they contain; construct wells display which DNA parts must be added to them. The parts already present in the wells at the time of a given part’s addition are displayed in bold. The route of each pipette tip is outlined by arrows: starting at the tip rack and then collecting the relevant DNA part solution from a source well, dispensing it to the construct wells and finally being discarded into the waste. (C) If the series of DVK additions is optimized, three pipette tips can be used instead of four without the risk of contamination.

However, the need for washing or changing the tip after each DNA part addition can be avoided. Suppose that at some point in the robot’s program a single tip has already distributed a certain DNA part to several wells and now proceeds to the next well. If none of the previously visited wells contain any DNA parts that have not been added to the next well earlier in the robot’s program, there is no risk of contaminating the next well by any DNA part that would not already be present there. Figure 1 showcases the minimization of pipette tip consumption for a simple example of parallel DNA assembly of a pair of two-part constructs following this principle.

Therefore, for every DNA part, the order in which it is delivered to the construct well where it is required can be optimized so as to minimize the number of pipette tips needed to perform all of its additions without risking contamination. This calls for an algorithm that receives an input describing which DNA parts have to be added to which construct wells and produces a sequence of pipette commands such that for each DNA part involved the order of actions is optimized, thereby minimizing the pipette tip consumption of the liquid-handling robot.

In this paper, we propose such an algorithm based on the capacitated vehicle routing problem (CVRP) [9]. This pipette tip-saving strategy was implemented in a Python package, which can be integrated into existing DNA assembly automation pipelines. We also present the results of testing our algorithm’s performance with randomly generated and real-life DNA assembly scenarios.

## 2. Materials and methods

The results in this paper were produced on a server with 2x Intel^®^ Xeon^®^ CPU E5-2670 2.60GHz processors with 128 GB RAM, running on Ubuntu 18.04 LTS. The algorithm was implemented in Python 3.8. The Python packages numpy 1.17, scipy 1.6, pandas 0.25, tspy 0.1.1, matplotlib 3.3 and ortools 9.1 were used. When integrating the algorithms into the DNA-BOT [2] and MoClo [11] assembly pipelines, the packages opentrons 3.21 and pyyaml 5.4 were additionally used.

## 3. Results

### 3.1 The capacitated vehicle routing problem

Let us define the CVRP in order to then show how its linear programming (LP) formulation can be modified to describe the pipette tip-saving problem. Essentially, the vehicle routing problem can be described as follows. There is a ‘depot’, where ‘goods’ are stored, and ‘customers’, to whom the goods must be delivered. The customers are connected among themselves and to the depot by ‘roads’, each of which has a ‘cost’ assigned to it. The objective is to use a fleet of ‘vehicles’, which can travel the roads, to deliver the goods to all customers while minimizing the sum of the costs of all roads that are traveled by the vehicles, which is the problem’s objective function. The constraints are that the number of vehicles in the fleet is fixed and that every vehicle must start and end its journey at the depot. In the CVRP, it is additionally specified that each vehicle can only carry an amount of goods that does not exceed the ‘vehicle capacity’.

The road network can be represented as a graph $G=(V,E)$, where customers are nodes $v_1, v_2, \ldots, v_n \in V$ with the depot indexed as the node $v_0 \in V$. The roads between them are edges $\{e_{ij}\ |\ 0 \leq i,j \leq n\}$ constituting the set *E*, where *e*_*ij*_ is the edge from node *v*_*i*_ to node *v*_*j*_ (note that *e*_*ij*_ and *e*_*ji*_ are two distinct edges) and the cost of the edge *e*_*ij*_ is given by the number $cost(e_{ij})=c_{ij}$. Therefore, going from the customer *v*_*i*_ to the customer *v*_*j*_ corresponds to traveling the edge *e*_*ij*_ and is associated with the cost *c*_*ij*_. The fleet size can be expressed as *K* and the vehicle capacity as *κ*. Finally, let us have variables $\{x_{ij}\ |\ 0 \leq i,j \leq n\}$, where $x_{ij}=1$ if the edge *e*_*ij*_ is traveled by any vehicle in the fleet and $x_{ij}=0$ otherwise. Then, the objective of the CVRP is to determine the values of $\{x_{ij}\}$ that minimize $C=\sum x_{ij}c_{ij}$ subject to linear constraints defined on *x*_*ij*_, *K* and *κ* [9]. The exact LP formulation of the CVRP is provided and explained in Section 1.1 of the Supplementary Text.

### 3.2 Casting the pipette tip-saving problem as a modified CVRP

The problem of pipette tip-efficient delivery of DNA parts to the wells that need them can be cast as a slightly modified CVRP.

Let us consider a numbered list outlining the order in which the parts are delivered. For the part number *h* in this list, let us construct a graph $G_h=(V,E)$ analogous to that for the CVRP. An aliquot of the liquid solution containing the DNA part being distributed corresponds to a ‘good’ that needs to be delivered to a ‘customer’ node *v*_*i*_, where each ‘customer’ is a well where a DNA assembly mix that includes the part in question is prepared. Meanwhile, every pipette tip used corresponds to a ‘vehicle’, delivering the DNA part solution (‘goods’) to the construct wells (‘customers’). As any pipette tip must start its journey at the DNA part solution source and end it at the waste bin, let us denote both these destinations as a single ‘depot’ node *v*_0_, from which each ‘vehicle’ trajectory must originate and where it must also end. As a single pipette tip’s volume is finite, there is a maximum number of wells it can serve, which defines its capacity *κ*.

Finally, the edges correspond to pipette transitions between wells, while their costs describe potential contamination events. Contamination may arise when the same pipette tip first visits a well containing a certain DNA part (one of the *h* − 1 DNA parts already added to the mixes requiring them) and then goes to a new well which does not have it, hence the latter well is contaminated by this part. More formally, for every well *v*_*a*_ we can write down a set $P^h_a$ listing all DNA parts present by the time the robot proceeds to distribute DNA part number *h*. If well *v*_*i*_ has been visited by the same tip before well *v*_*j*_ is visited, then *v*_*j*_ would be contaminated if $P^h_i \not \subseteq P^h_j$. Conversely, there would be no contamination if $P^h_i \subseteq P^h_j$, as this implies that all parts present in *v*_*i*_ are already found in *v*_*j*_. Thus, even if some traces of DNA stay on the tip after contact with the solution in *v*_*i*_, this cannot introduce any new DNA parts to *v*_*j*_.

To keep track of all such contaminations, let there be an edge $e_{ij} \in E$ between any two nodes $v_i,v_j \in V$, such that $cost(e_{ij})=1$ if $P^h_i \not \subseteq P^h_j$ and $cost(e_{ij})=0$ otherwise (the ‘depot’ is also connected to every well, and any edge from or to it—$e_{0i}$ or $e_{i0}$ respectively—has cost 0 by definition, since collecting the part solution from the source or trashing the tip is irrelevant to cross-contamination between the wells). As long as a single tip’s route includes only zero-cost edges, for any well $v_{\alpha_l}$ we know that none of the previously visited wells $v_{\alpha_1},v_{\alpha_2}, \ldots, v_{\alpha_{l-1}}$ contain DNA parts not present in $v_{\alpha_l}$ already: indeed, since $cost(e_{\alpha_{l-1} \alpha_{l}})=0$, we have $P^h_{\alpha_{l-1}} \subseteq P^h_{\alpha_{l}}$; then, since $cost(e_{\alpha_{l-2} \alpha_{l-1}})=0$, $P^h_{\alpha_{l-2}} \subseteq P^h_{\alpha_{l-1}} \subseteq P^h_{\alpha_{l}}$. We can proceed by induction until we obtain $P^h_{\alpha_1} \subseteq P^h_{\alpha_2} \subseteq \ldots \subseteq P_{\alpha_l}$, which excludes the contamination condition.

The objective of the pipette tip-saving problem is to find the variables $\{x_{ij}\}$ (where, as previously, *x*_*ij*_ indicates whether the edge *e*_*ij*_ is traveled by any of the ‘vehicles’) such that the number of tips (‘vehicles’) used is minimized while cross-contamination is avoided and no tip serves more wells than its capacity *κ* allows. This means that our problem’s formulation is identical to the CVRP, except for two changes. First, the total cost of all the traveled edges *C* is fixed at zero to avoid contamination; second, the ‘fleet size’ (number of pipette tips used) *K* is not a fixed value, but the objective function to be minimized (see Supplementary Text, Section 1.2 for the exact formulation).

### 3.3 Solving the pipette tip-saving problem as a linear program

The advantage of expressing our pipette tip-saving problem as a linear program (LP) is that LP constitutes a well-studied class of problems, for which numerous powerful numerical solvers have been developed. Therefore, the LP formulation of the problem of distributing a single DNA part to the construct wells can be leveraged to propose an algorithm that makes use of an LP solver to minimize pipette tip consumption over the whole procedure of preparing DNA assembly master mixes. We implemented such an algorithm in Python, solving the arising LP problems with the CP-SAT solver from the optimization package OR-Tools [10].

Briefly, the algorithm is provided with the composition of the DNA constructs to be assembled, as well as the location of the wells where the corresponding mixes should be prepared, and of the sources of DNA part solutions. From this input, it determines a list describing which DNA part will be distributed first across all the wells that require it, which part will be delivered second, and so on. Now, for every part in this list, an LP problem is solved, which allows to determine the best way of delivering it. Reaching the end of the part list means that we have obtained a sequence of liquid-handler commands which enables the contamination-free distribution of all parts using a reduced number of pipette tips.

Notably, our LP problem is only concerned with optimizing the pipette’s actions as it distributes a given DNA part. Meanwhile, the order in which DNA parts themselves are distributed (which part is the first to be dispensed to all wells that need it, which part is dispensed to all wells that require it second, which part is the third, etc.) must be pre-determined. Currently, we either determine the DNA part sequence randomly or distribute the parts in the order they are encountered in the list of constructs to be prepared, which the algorithm receives as input. In the latter case, we start by distributing the DNA part that is found first in the part list of the first construct we read. We then proceed down the input list of constructs and their compositions, adding any newly encountered DNA part to the end of the sequence that defines the DNA part order (see Supplementary Text, Section 2).

**Figure 2.**
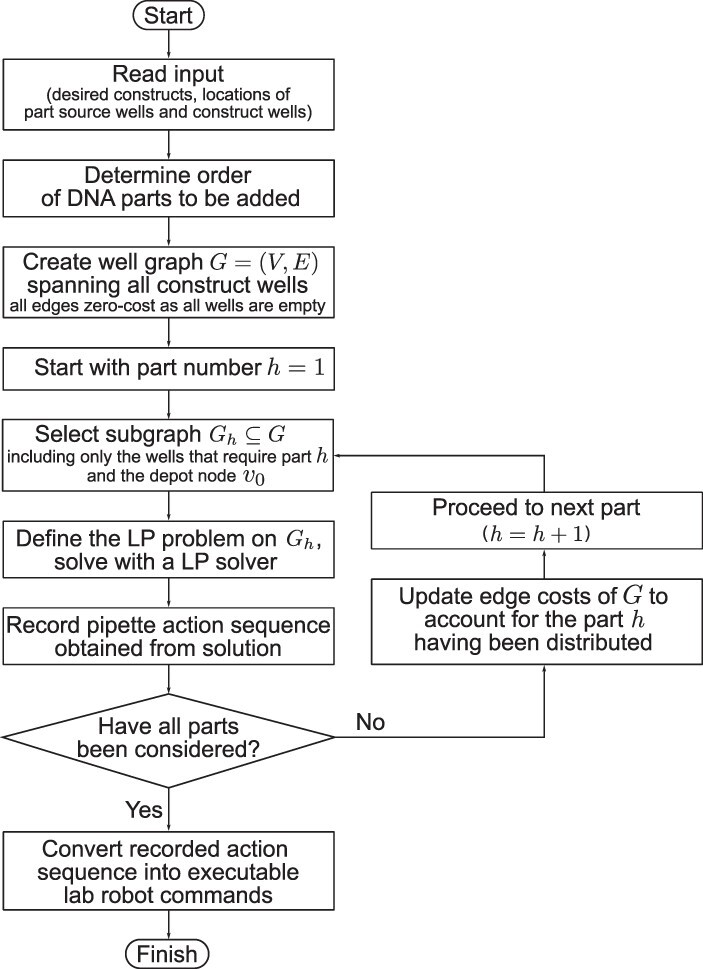
Flowchart describing the proposed pipette tip consumption minimization algorithm.

**Figure 3.**
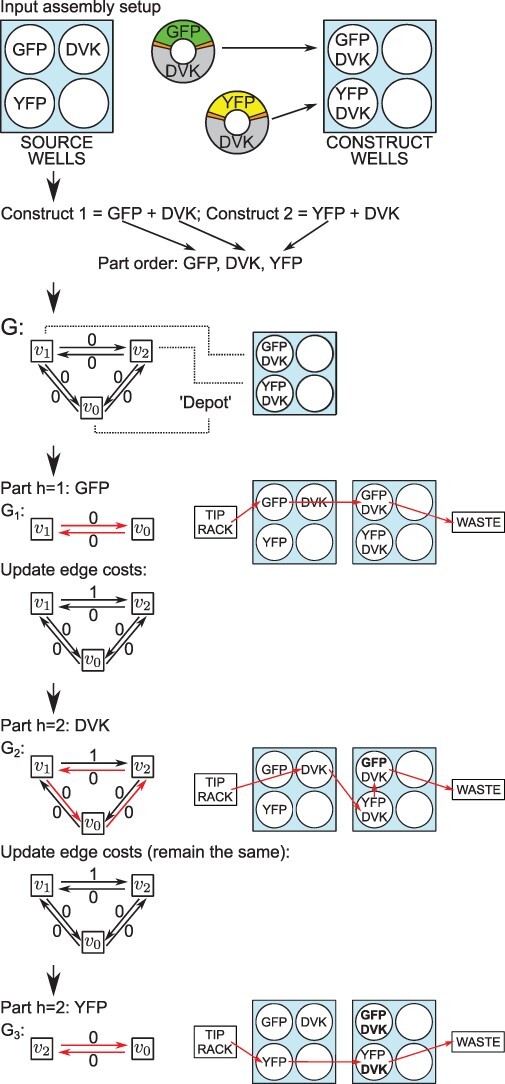
The algorithm applied to an example assembly setup, yielding the optimized series of robot commands, which uses only three tips to perform four part additions. This is the same solution as in the optimized case in Figure 1C. The edges traveled by a pipette tip in the LP problem’s solution are displayed in red. Note how the cost-one edge is avoided in *G*_2_, thereby preventing contamination. Also observe how after adding GFP we update $cost(e_{12})$, as GFP from *v*_1_ can now contaminate empty *v*_2_. Formally, it can be said that after adding the part *h* = 1 we have $P^2_1=\{DVK\}\not\subseteq\varnothing=P^2_2$, hence $cost(e_{12})=1$. Meanwhile, $cost(e_{21})=0$ even after DVK is added to *v*_2_, as DVK is now present in both construct wells, so the non-contamination condition $P^3_2=\{DVK\}\subseteq\{GFP,DVK\}=P^3_1$ is still satisfied. Thus, DVK contained in *v*_2_ cannot contaminate *v*_1_, as this latter well contains DVK anyway.

The algorithm is described in more detail in Figure 2, while Figure 3 demonstrates our algorithm in action for the example considered in the introduction.

### 3.4 Testing the pipette tip-saving algorithm’s performance

**Figure 4.**
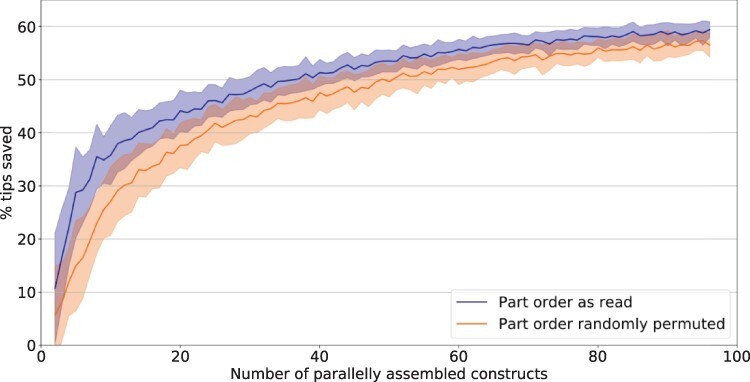
Results of performance testing with randomly generated Start–Stop assembly inputs, with the number of parallelly assembled constructs varying from 2 to 96. For each number of constructs considered, the mean percentage of tips saved by our algorithm for *N* = 50 different random inputs is shown. The shading shows the range of percentages within the standard deviation from the mean.

To evaluate the performance of our algorithm, we implemented it in Python and tested it on randomly generated inputs for the Start–Stop DNA assembly protocol [8]. Each assembled gene we considered in our tests consists of four parts: a promoter, a ribosome-binding site (RBS), a coding sequence (CDS) and a terminator. In this example, a construct is generated by picking one of six possible promoters, one of four RBSs, one of three CDSs and one of three terminators, reflecting the sizes of DNA part libraries used in a past experiment in our labs. For every number of constructs from 2 to 96, we generated 50 random assembly scenarios with this many random constructs assembled in parallel (see Start-Stop_Assembly_Random_Inputs.csv and Section 3 of the Supplementary Text). For each of these sets of 50 optimizations, the mean percentage of pipette tips saved relative to the case where each part addition is performed with a fresh tip was calculated and plotted in Figure 4 (see Random_Input_Testing_Results.xlsx and Supplementary Text, Section 4).

To demonstrate that the algorithm can save tips in real cases as well as for randomly generated ones, we turned to the packages DNA-BOT [2] and ‘OT2 Modular Cloning (MoClo) and Transformation in *E. coli* Workflow’ [11], which automate DNA assembly of the BASIC and MoClo standards, respectively, for the Opentron OT-2 liquid-handling platform. They contain test examples of known parallel DNA assembly scenarios, which can be used to assess the automation pipelines’ accuracy and efficiency—the complete sets of inputs can be found in these packages’ code repositories [2; 11]. The results of optimizing the pipette movements for the situations described by them are displayed in Table 1 and 2.

**Table 1.** Results of performance testing with BASIC assembly test inputs

	% tips saved
Part order	BASIC, 5 parts (max. 440 tips^a^)
As read	49.55
Random	49.77

a Pipette tips consumption when a fresh tip is used for each part addition.

**Table 2.** Results of performance testing with MoClo assembly test inputs. The example constructs were split by the numbers of parts they comprise

	% tips saved		
	MoClo	MoClo	MoClo
	2 parts	5 parts	8 parts
Part order	(max. 48 tips^a^)	(max. 120 tips^a^)	(max. 192 tips^a^)
As read	31.25	50.00	54.69
Random	25.0	26.67	35.94

a Pipette tips consumption when a fresh tip is used for each part addition.

To assess the effect of the order of DNA parts, for every input the optimization was performed both with a randomly permuted DNA part order and with the parts added in the order of their appearance in the input list of DNA constructs to be made (from the part first encountered in the list of constructs to the last one—see Supplementary Text, Section 2). Hence, Figure 4, Table 1 and Table 2 each display two different sets of results, labeled ‘part order randomly permuted’ or ‘part order as read’, respectively.

With more than half of pipette tips saved for the randomly generated inputs describing high numbers of parallelly prepared Start–Stop assembly constructs, the results demonstrate the algorithm’s potential to significantly improve resource efficiency for parallel DNA assembly under various standards. As the number of constructs assembled in parallel grows, the tip savings rise. A likely explanation for this is that with greater input sizes, a single part is found in more constructs being prepared, and solving the CVRP-like problem on a larger subgraph can potentially yield longer chains of wells which the same pipette tip can visit. Therefore, the benefits of algorithmic pipette tip uptake optimization could be expected to improve even more as synthetic biology research shifts toward larger-scale experiments and high-throughput screening [12; 13]. To confirm our findings, we also ran our algorithm for the same inputs using the GUROBI^®^ optimizer (Python package gurobipy 9.1) [14], which our package can employ instead of OR-Tools CP-SAT as an alternative LP solver. Indeed, very similar results were obtained.

While even with DNA parts listed in random order considerable optimization was achieved, using the part order as read from the input (Supplementary Text, Section 2) resulted in greater pipette tip savings in all scenarios considered except for BASIC assembly. Therefore, there may exist patterns in how the part order is defined that on average increase pipette tip savings. In the future, identifying and exploiting such input patterns alongside our proposed LP-based optimization of actions to distribute an individual part could yield an even more powerful pipette tip-saving method.

## 4. Discussion

We propose an LP formulation of the problem of saving pipette tips while delivering a single part to the wells where one-pot DNA assembly reactions are being performed in parallel. In addition to this, we provide performance test results for both randomly generated and real-life examples, demonstrating that algorithms leveraging this formulation in conjunction with a linear programming solver can be used to significantly reduce pipette tip consumption.

The resultant decrease in pipette tip uptake brings about several benefits. First, fewer used plastic tips are thrown away in a single DNA assembly run, which makes laboratory research more environmentally sustainable. Second, if a robotic liquid-handler’s pipette changes tips less often, it has to make fewer visits to the pipette tip rack, where it picks up new tips, and to the waste bin, where the old ones are discarded. These pieces of labware occupy separate slots on the robot’s platform, so traveling to them takes more time than simply moving between several wells of the same plate. Consequently, delivering the DNA part solution to several construct wells with a single tip also makes automated DNA assembly faster. For the DNA-BOT package’s test case of preparing 88 5-part BASIC assembly constructs [2], we estimate that the adoption of our algorithm can reduce the protocol’s execution time by as much as 73 minutes or $48\%$ of the total time required to distribute all DNA parts to the construct wells. Finally, the economical benefit of improving the DNA assembly protocol can be calculated by considering the decreased spending on consumables (i.e. pipette tips). For the same BASIC assembly test case, we evaluate the cost savings provided by our algorithms to amount to $\$ 6.54$ or $5.27\%$ of the cost of all consumables per one run of this large-scale parallel DNA assembly (see Supplementary Text, Section 3).

Underlying this potentially powerful DNA assembly optimization strategy is the combination of a robotic liquid-handling platform and automatic process optimization. Apart from being slower than a laboratory robot, a human worker with a conventional pipette can only collect a specified volume of solution and then dispense it all into the desired well, while distributing exact volume fractions across several destinations is typically not done manually. On the contrary, a robotic liquid handler’s regulated pumps allow to perform this routinely. Consequently, having a robotic liquid handler follow simple pipetting protocols tailored for a human worker does not result in optimal procedure execution, leaving open the avenues to realize the full potential of laboratory automation. Meanwhile, manually optimizing the laboratory protocol instead of using automated algorithmic solutions would be a repetitive procedure required for every new assembly setup, becoming especially tedious and challenging for large-scale assemblies with dozens of potential contaminants to keep track of.

Our algorithm currently focuses on the lowest level of protocol optimization, as it manages individual pipette actions for the distribution of a single part at a time. It is also not restricted to any single one-pot DNA assembly method. This allows to achieve optimization of pipette tip consumption for a variety of DNA assembly automation pipelines following different standards, as well as to combine our algorithms with higher-level optimization strategies (that can determine which DNA parts should be used and what is the best sequence of steps to combine them all [4; 5]) to achieve a truly multimodal optimization of automated DNA assembly.

The proposed algorithm’s implementation in Python code is available via our open-access GitHub repository pipette_opt [15]. Our package offers Application Programming Interface solutions which allow users to incorporate our algorithm for pipette tip consumption minimization into existing DNA assembly pipelines for the Opentrons OT-2 liquid handler, namely DNA-BOT [2] and ‘OT2 Modular Cloning (MoClo) and Transformation in *E. coli* Workflow’ [11]. Upon downloading pipette_opt, minor changes to the original code of a DNA assembly automation pipeline should be made according to the instructions provided. Following this one-off code modification, the user can leverage our algorithm to determine the optimized sequence of pipette actions during the DNA part distribution step and translate it into Opentrons OT-2 commands, which are inserted into the overall program generated by the main Opentrons automation pipeline. When the protocols generated by the modified pipelines were tested *in silico* using the Opentrons OT-2 simulation feature, we could confirm that the liquid-handling robot’s optimized actions are accompanied by a significant reduction in pipette tip consumption.

## Supplementary Material

ysac004_Supp

## Data Availability

The MoClo and BASIC assembly test inputs are available at https://github.com/DAMPLAB/OT2-MoClo-Transformation-Ecoli and https://doi.org/10.1093/synbio/ysaa010, respectively. The random Start–Stop assembly testing inputs and the results of testing the algorithm for all assemblies are available in the article and its online supplementary material.

## Material availability

The Python package is available at: https://github.com/KSechkar/pipette_opt.
