# Supplementary material for: A linear programming-based strategy to save pipette tips in automated DNA assembly
Source: Synth Biol (Oxf). 2022 Apr 11;7(1):ysac004. doi: 10.1093/synbio/ysac004 (PMC9074407; doi:10.1093/synbio/ysac004)
Supplement: ysac004_Supp [file ysac004_supp.zip › Supplementary Text.pdf]

# Supplementary Text:

# A linear programming-based strategy to save pipette tips in automated DNA assembly

Kirill Sechkar[1] Zoltan A Tuza[1] Guy-Bart Stan[1,*]

[1]Department of Bioengineering, Imperial College London,

South Kensington Campus, SW7 2AZ, London, UK

[*]Corresponding author. Email: g.stan@imperial.ac.uk

# Contents

**1 Linear Programming Problem Formulation** **3**

1.1 Formulation of the Capacitated Vehicle Routing Problem . . . . . . . . . . 3

1.2 Formulation of the pipette tip-saving Problem . . . . . . . . . . . . . . . . . 7

**2 Determination of the order of DNA part distribution** **8**

**3 Estimation of time and cost savings** **9**

**4 Legend to `Start-Stop_Assembly_Random_Inputs.csv`** **12**

**5 Legend to `Random_Input_Testing_Results.xlsx`** **12**

**6 Legend to `Random_Input_Testing_Results-Individual_Runs.xlsx`** **13**

# 1 Linear Programming Problem Formulation

## 1.1 Formulation of the Capacitated Vehicle Routing Problem

As discussed, our LP-based algorithm for reducing the pipette tip consumption uses a modified version of the Capacitiated Vehicle Routing Problem (CVRP).

Briefly, the objective of the CVRP is to deliver goods from the depot to all customers using a given number of vehicles that carry the goods, so as to minimize the total cost of all roads that are travelled by the vehicles. Its definition in terms of graph theory is as follows. In a "road network" graph $G = (V, E)$, the nodes $v_1, v_2 \ldots, v_n \in V$ represent the customers ($V$ also includes a special "depot" well $v_0$). Meanwhile, the roads are given by the edges of the set $E = \{e_{ij} \mid 0 \leq i, j \leq n\}$, where $e_{ij}$ is the edge from node $v_i$ to node $v_j$ and $cost(e_{ij}) = c_{ij}$ is the cost of travelling the road from the customer $v_i$ to the customer $v_j$. A fleet of $K$ cars is disposable, each of which must start and terminate its journey at the depot $v_0$ and can serve at most $\kappa$ customers due to vehicle capacity limitations.

The *vehicle flow model* of the CVRP equates determining the vehicles' routes to finding a set of variables $\{x_{ij} \mid 0 \leq i, j \leq n\}$ that indicate which edges have been traversed by some vehicle: $x_{ij} = 1$ if $e_{ij}$ belongs to any of the vehicles' routes and $x_{ij} = 0$ otherwise. Therefore, by introducing integer dummy variables $u_1, u_2 \ldots, u_n$, we can put down a strict LP formulation of the CVRP [6]:

$$\text{minimize } C = \sum_{i=0}^{n} \sum_{i=0}^{n} x_{ij} c_{ij}$$

by determining $\{x_{ij}\}$ and $\{u_i\}$ such that

$$\sum_{i=1}^{n} x_{ij} = 1, \quad j = 1...n \qquad \text{(Constraint 1.1a)}$$

$$\sum_{j=1}^{n} x_{ij} = 1, \quad i = 1...n \qquad \text{(Constraint 1.1b)}$$

$$\sum_{i=1}^{n} x_{0i} = K \qquad \text{(Constraint 1.2a)}$$

$$\sum_{i=1}^{n} x_{i0} = K \qquad \text{(Constraint 1.2b)}$$

$$u_j - u_i \geq 1 - \kappa(1 - x_{ij}) \;\; 1 \leq i, j \leq n, \; i \neq j \qquad \text{(Constraint 1.3a)}$$

$$0 \leq u_i \leq \kappa - 1 \;\; 1 \leq i \leq n \qquad \text{(Constraint 1.3b)}$$

**The objective function** in this formulation is indeed the cost of all roads travelled by the vehicles. For every pair $(i, j)$ considered, its contribution to the sum is equal to $0 \times c_{ij} = 0$ if $x_{ij} = 0$, and $1 \times c_{ij} = c_{ij}$ if $x_{ij} = 1$. Therefore, as all possible $(i, j)$ pairs are considered, $C$ is the sum of the costs of all edges whose associated variable $x_{ij}$ is equal to 1. These by definition are exclusively the edges travelled by any of the vehicles.

**Constraint 1.1** means that every customer node is visited exactly once – as every customer must be served, having a node that is not visited by any vehicle at all is inadmissible; meanwhile, there is no need to come to a customer again once they have been served, hence multiple visits also being unfavorable. Thus, for any customer $v_\alpha$, exactly one incoming edge is selected: otherwise, the sum in Constraint 1.1a would amount to 0 if $x_{i\alpha} = 0 \; \forall i = 1 \ldots i$, or would be at least 2 if there are at least two different indices $i_1 \neq i_2$ such that $x_{i_1\alpha} = x_{i_2\alpha} = 1$. A similar argument can explain how Constraint 1.1b implies that exactly one outgoing edge is travelled by a vehicle. Together, these constraints mean that a single vehicle first arrives at the customer node via the only edge $e_{i\alpha}$ for which $x_{i\alpha} = 1$, then leaves it via the one edge $e_{\alpha j}$ such that $x_{\alpha j} = 1$.

**Constraint 1.2** defines the vehicle fleet size. As every vehicle must depart from and arrive to the depot node $v_0$ exactly once, Constraint 1.2a sets the number of selected edges coming to $v_0$ as exactly $K$, while Constraint 1.2b sets the number of selected edges. Similarly to Constraint 1.1, selecting fewer edges means the sum in question is less than $K$, while selecting too many means that the sum exceeds $K$.

**Constraint 1.3** finalizes the formulation by introducing auxiliary integer variables $u_1 \dots u_n$ (see [6]) to address the following issues. First, the CVRP requires to account for vehicle capacity, which does not appear in Constraints 1.1-1.2. Second, by finding the $x_{ij}$ values, we merely determine if a given edge is included into the set of selected roads $\{e_{ij} | x_{ij} = 1\}$. These chosen roads form paths on the road network graph. Due to Constraint 1.1, every path is a cycle: since every customer node has exactly one incoming and exactly one outgoing selected edge, no node can be terminal in a non-cyclical path (this would imply that no outgoing edge is selected for it). Meanwhile, Constraint 1.2 means that $K$ of these cycles go through the depot node $v_0$. However, these two constraints do not forbid the selected edges from forming a cycle that does not go through $v_0$. Yet, such cycles do not match the definition of a vehicle route, because all vehicles must start and finish their journey at the depot.

Let us consider Constraint 1.3a. If the road between the customers $v_i$ and $v_j$ is not travelled by any vehicle ($x_{ij} = 0$), the right-hand side amounts to $1 - \kappa$. Then, given that $u_j \geq 0$ and $u_i \leq \kappa - 1$ due to Constraint 1.3b, we can observe that

$$u_j - u_i \geq 0 - (\kappa - 1) = 1 - \kappa = 1 - \kappa(1 - 0) = 1 - \kappa(1 - x_{ij}).$$

Hence, if no vehicle travels the road between the two customers, Condition 1.3a is always upheld regardless of $u_i$ and $u_j$, provided they satisfy Condition 1.3b. Conversely, if $x_{ij} = 1$,

Condition 1.3a imposes that

$$u_j - u_i \geq 1 - \kappa(1 - x_{ij}) = 1 - \kappa(1 - 1) = 1 \Leftrightarrow u_j > u_i.$$

Let us now show why this means that the same vehicle cannot visit $L > \kappa$ customers. Even if $v_{\beta_1}$, the first customer visited by the vehicle after the depot, is assigned with the minimum possible value $u_{\beta_1} = 0$, we will have $u_{\beta_2} > u_{\beta_1} = 0 \Rightarrow u_{\beta_2} \geq 1$ for the next customer on the route ($v_{\beta_2}$). Likewise, for the following customer we will have $u_{\beta_3} \geq u_{\beta_2} \geq 1 \Rightarrow u_{\beta_3} \geq 2$. By the time the $L$th customer is visited, we will thus have $u_{\beta_L} \geq L - 1$, where $L > \kappa \Rightarrow L - 1 > \kappa - 1$. Consequently, Constraint 1.3b is violated.

Selecting cycles that do not go through the depot is similarly forbidden. In such a cycle $v_{\gamma_1}, v_{\gamma_2}, \ldots, v_{\gamma_M}, v_{\gamma_1}$ with $\gamma_i \neq 0 \; \forall i$, Constraint 1.3a leads to a contradiction. Namely, it implies that $u_{\gamma_1} < u_{\gamma_2} < \ldots u_{\gamma_M} < u_{\gamma_1} \Rightarrow u_{\gamma_1} < u_{\gamma_1}$.

Meanwhile, vehicle routes that go through the depot and span $N \leq \kappa$ customers are still permitted. Let us simply start with $u_{\delta_1} = 0$ for the customer $v_{\delta_1}$ visited straight after the depot, and increase the $u$ value by 1 for each next customer on the route. Thus, for two consecutive customers on the route, $v_{\delta_i}$ and $v_{\delta_{i+1}}$, we always have $u_{\delta_{i+1}} = u_{\delta_i} + 1 \Leftrightarrow u_{\delta_{i+1}} > u_{\delta_i}$, in line with Constraint 1.3a. Furthermore, the route's final customer is assigned with $u_{\delta_N} = N - 1 \leq \kappa - 1$, so all the values $u_{\delta_1}, u_{\delta_2}, \ldots, u_{\delta_N}$ belong to the range between 0 and $\kappa - 1$, thereby satisfying Constraint 1.3b.

**The obtained values** $x_{ij}$ always allow us to uniquely determine every vehicle's journey. The start of a single vehicle's journey is marked by a variable $x_{0\delta_1} = 1$: following the road $e_{0\delta_1}$, the vehicle arrives to the customer node $v_{\delta_1}$. Due to Constraint 1b, there is only one non-zero variable $x_{\delta_1\delta_2} = 1$ among those that leave the considered customer node $v_{\delta_1}$. Hence, the next node on the route is $v_{\delta_2}$. Similarly, at $v_{\delta_2}$ the next customer is revealed by the only non-zero variable $x_{\delta_2\delta_3} = 1$. The vehicle proceeds in this manner

until for the route's final customer $v_{\delta_N}$ we have $x_{\delta_N 0} = 1$.

## 1.2 Formulation of the pipette tip-saving Problem

For our pipette tip-saving problem, we have a similar graph $G_h = (V, E)$ describing the wells to which the DNA part number $h$ in the sequence of parts to be delivered. The "goods" are the DNA part solution, the "customers" are the construct wells (nodes $v_1, v2 \ldots, v_n \in V$), each "vehicle" is a single pipette tip and $cost(e_{ij}) = c_{ij} = 0$ if the same pipette tip can proceed from the well $v_i$ to the well $v_j$ without any cross-contamination. The objective is to deliver a DNA part to all customers requiring it by using as few vehicles as possible. Therefore, the total cost of the vehicle routes is constant at $C = 0$ (including a cost-one edge into any tip's route implies a tip change in the middle of the way, which is a contradiction), while the objective is to minimize $K$, the number of vehicles. This "swapping" of the objective function and Constraint 2, while keeping all other constraints in place, yields the following formulation:

$$\text{minimize } K = \sum_i x_{0i}$$

by determining $\{x_{ij}\}$ and $\{u_i\}$ such that

$$\sum_i x_{ij} = 1, \quad j = 1...n \qquad \text{(Constraint 2.1a)}$$

$$\sum_j x_{ij} = 1, \quad i = 1...n \qquad \text{(Constraint 2.1b)}$$

$$\sum_i \sum_j x_{ij} c_{ij} = C = 0 \qquad \text{(Constraint 2.2)}$$

$$u_j - u_i \geq 1 - \kappa(1 - x_{ij}) \;\; 1 \leq i, j \leq n, \; i \neq j \qquad \text{(Constraint 2.3a)}$$

$$0 \leq u_i \leq \kappa - 1 \;\; 1 \leq i \leq n \qquad \text{(Constraint 2.3b)}$$

The obtained $\{x_{ij}\}$ values reveal the route of every tip as it travels between construct wells, similarly to how the route of every vehicle can be determined from $\{x_{ij}\}$ in the CVRP. Knowing the sequence of wells visited by a single tip, it is easy to write down the commands for the liquid-handler, ordering it to first collect a fresh pipette tip and the relevant DNA part solution, then deliver aliquots of it to the construct wells on the tip's itinerary, and finally discard the tip. Together, these command sequences for every pipette tip yield the optimized lab robot program which enables the delivery of the DNA part in question to all construct wells that need it.

## 2 Determination of the order of DNA part distribution

As every DNA part is assigned its own LP problem, solving which can save pipette tips, the decision which DNA part will be distributed (to all wells that require it) first, which part the second and so on, is not subject to optimization by the LP solver. Instead, it is determined by an ordered list $\lambda$ that includes all of the DNA parts.

How can the order of DNA parts be determined in $\lambda$? During algorithm testing, the sequence of DNA parts was random, or alternatively, the parts were given in the order they were encountered in the input. While the former strategy is easy to understand, let us strictly define the latter.

The input to our program that implements the tip-optimizing algorithm is a tuple $\Omega = (\omega_1, \omega_2 \ldots, \omega_n)$, where each entry $\omega_i$ (the index signifies that it is found in the the $i$th position in the tuple) is the list of DNA parts comprizing a single DNA construct to be assembled – $p_j$ is the $j$th DNA part in this list. Therefore, $\lambda$ is constructed by "reading

through" $\Omega$, as given by Algorithm 1: first, it records into $\lambda$ all parts found in $\omega_1$; then, it considers $\omega_2 \in \Omega$ and records into $\lambda$ all parts found in $\omega_2$ that have not been recorded in $\lambda$ already; then the program proceeds to record all previously unrecorded parts found in $\omega_3$ and so on until the end of $\Omega$ is reached.

**Algorithm 1** Reading $\lambda$ from $\Omega$

```
λ = ( )
for ω_i in Ω do
    for p_j in ω_i do
        if p_j ∉ λ then
            append p_j to λ
```

As mentioned in Discusson, optimization of the order of parts in $\lambda$ may further reduce pipette tip consumption in addition to the optimization provided by our algorithm, which opens up a promizing venue for improving the algorithm. As it is impossible to determine the pipette tip savings for every possible $\lambda$ without explicitly running the algorithm with it, such reorderings of $\lambda$ are likely to be heuristic, rather than strictly optimal.

# 3 Estimation of time and cost savings

Besides avoiding excessive plastic waste, a reduction in pipette tip use can also improve the time-efficiency of automated DNA assembly. Both the rack with new pipette tips and the waste bin, where the used tips must be discarded, occupy separate labware slots on the lab robot's platform. Therefore, the time the pipette spends travelling between different slots to change the tip is coniderably greater than the time to travel between two wells of the same 96-well plate if the same tip is kept.

To estimate the time required to change a pipette tip, we used the protocol timer

feature of the opentrons Python package, which can simulate the execution of a program by the Opentrons OT-2 robot to estimate the time it would take in real life. A script for transferring 1 $\mu L$ of solution to each of the 96 wells of a "construct" plate from a well on the "DNA part source" plate was written and simulated. The results were then compared with an alternative script, which instead filled the plate on a "two-by-two" basis by collecting 2 $\mu L$ of solution and then dispensing it to two wells on the plate with the same tip. Therefore, the second protocol involved 48 fewer tip changes. The simulation and timing of separate scripts was necessary due to the protocol timer feature becoming available only from the 4.6.0 version of the opentrons package [2]. Meanwhile, both DNA-BOT [5] and "OT2 Modular Cloning (MoClo) and Transformation in *E. coli* Workflow" [4] were written with opentrons 3.x in mind. Thus, the commands they produce cannot be timed as they are being simulated.

The protocol that reused the tips was estimated to run 14 *min* faster, which signifies that around 17.5 *sec* is lost on every additional tip change. Consequently, the algorithm saves 49.55% of all tips (i.e., 218 out of 440) in the DNA-BOT pipeline's test case of assembling 88 5-part constructs [5]. This amounts to

$$17.5 \frac{sec}{tip} \times 218\ tips\ saved = 3815\ sec \approx 63.6\ min.$$

For this example, we also directly timed the DNA part distribution step by simulation, and then compared it with the result for optimized DNA part distribution. Algorithmic optimization reduced the part distribution time by 73 *min* (48%), taking it down from 152 *min* to 79 *min*. The difference between the estimated and the obtained value can be explained by the fact that the distance travelled to and from the tip rack is not always the same, especially when several racks of 96 tips, each in a different slot, are required to satisfy the procedure's demands. Moreover, the volumes aspirated or dispensed also vary

throughout the optimized protocol depending on how many wells a given tip serves, so the pipetting itself may not always be of the same duration. Additional possible sources of discrepancy include the use of air gaps to separate DNA part aliquots in our program, and the fact that the original pipeline mixes the added part with the well's contents by pipetting $3\mu L$ up and down. The script used to estimate all of the runtimes can be found in the GitHub repository `pipette_opt` [3], together with the implementation of our algorithm.

Saving pipette tips also implies that less money is spent on consumables for a single parallel DNA assembly array. Here, we estimate these savings based on the data provided in the DNA-BOT publication (representing the prices of consumables as of October 2019) [5], as this allows us to compare our obtained values with the total cost of automated DNA assembly evaluated by the authors. One Opentrons p10 tip was found to cost roughly \$ 0.03 in the publication, which is not too far from the cost of \$ 0.0315 per tip in an order of 1,000 tip rack refills as of January 2022 [1]. Thus, for the DNA-BOT package's test scenario that we consider, the economic benefit per one run of a large-scale parallel DNA assembly can be estimated to be

$$0.03\frac{\$}{tip} \times 218\ tips \approx \$\ 6.54.$$

A single run of DNA assembly with the DNA-BOT pipeline costs roughly \$ 1.41 (without considering the cost of competent cells that are later transformed with the assembled DNA constructs) [5]. Therefore, the percentage decrease in parallel DNA assembly costs is:

$$(\$\ 6.54) \div (1.41\frac{\$}{construct} \times 88\ constructs) \times 100\% \approx 5.27\%.$$

# 4 Legend to `Start-Stop_Assembly_Random_Inputs.csv`

As Discussed in Results and Methods, for each input size, from just 2 constructs to 96 different construct wells (i.e., the whole well plate), 50 Start-Stop assembly inputs were created, randomly picking one of 6 possible promoters, one of 6 RBSs, one of 3 CDS variants and one of 4 terminators.

In the abstract notation used by the algorithms, a DNA part is given by a pair $(i, j)$, where $i$ is the DNA part type (promoter, CDS, etc.) and $j$ specifies which species of this type it is – in our case, $i = 1$ stood for the promoter parts, $i = 2$ for RBS, $i = 3$ for CDS and $i = 4$ for the terminator. Therefore, each construct well in the file is a single line that has 4 entries, each of which is a pair of numbers encoding the part and its type.

Each input is terminated by a line saying "end of input" to separate it from the beginning of the next construct well plate description. The inputs for different numbers of wells are separated by a blank line.

This file can also be found in the GitHub repository that contains our algorithm's Python implementation [3], so that the results presented in the paper could be reproduced.

# 5 Legend to `Random_Input_Testing_Results.xlsx`

The optimization of a single input by an algorithm produced a single number, which describes how many pipette tips are required to perform parallel DNA assembly in the optimized case.

Means, medians and standard deviations of these values were taken across the 50 inputs for every number of wells considered (2 to 96) – these results are displayed in separate tabs of the workbook. The next three tabs also contain means, medians and standard

deviations of the percentages of tips saved by the algorithms, which are calculated using the formula:

$$\% \text{ tips saved } = \frac{(unoptimized\ no.\ tips\ used) - (optimized\ no.\ tips\ used)}{(unoptimized\ no.\ tips\ used)} \cdot 100\%.$$

# 6 Legend to `Random_Input_Testing_Results-Individual_Runs.xlsx`

In addition to the mean, median, and standard deviation calculated for all test inputs with the same construct number, we supply the individual pipette tip usage values for all optimized test cases. Each tab of the workbook contains the data for all cases that concern the same number of parallelly assembled constructs from 2 to 96. There are 50 columns, each for one of the 50 inputs considered. As for the rows, there are two in each tab, so each column contains the tip uptake (a) when the order of DNA part additions is determined randomly and (b) when the parts are listed in the order they are read from the input (see Section 2 of this Supplementary Text).

# References


[1] *20μL pipette tips - Opentrons*, `https://shop.opentrons.com/opentrons-20-l-tips-160-racks-800-refills/`, (29 january 2022, date last accessed).

[2] *opentrons/CHANGELOG.md at edge · Opentrons/opentrons*, `https://github.com/Opentrons/opentrons/blob/edge/CHANGELOG.md`, (29 january 2022, date last accessed).

[3] Zoltan A. Tuza Kirill Sechkar. *pipette_opt.* `https://github.com/KSechkar/pipette_opt` (15 march 2021, date last accessed).

[4] Marilene Pavan Rita Chen, Nicholas J. Emery and Samuel M.D. Oliveira. *Laboratory Protocol Automation: A Modular DNA Assembly and Bacterial Transformation Case Study*, pages 21–22. Bio-Design Automation Consortium, 2020.

[5] Marko Storch, Matthew C Haines, and Geoff S Baldwin. DNA-BOT: a low-cost, automated DNA assembly platform for synthetic biology. *Synthetic Biology*, 5(1), 07 2020. ysaa010.

[6] Paolo Toth and Daniele Vigo. *1. An Overview of Vehicle Routing Problems*, pages 1–26. Society for Industrial and Applied Mathematics, 2002.
